# Attitudes Toward Psychiatry Among Medical Students With a Psychiatric Major at Chongqing Medical University

**DOI:** 10.3389/fpsyt.2022.820060

**Published:** 2022-02-14

**Authors:** Xiao Li, Ming Ai, Xiaolu Chen, Yao Gan, Jinglan He, Yu Tian, Jian Zhai, Haoling Yang, Li Kuang

**Affiliations:** ^1^Department of Psychiatry, The First Affiliated Hospital of Chongqing Medical University, Chongqing, China; ^2^The First Branch, The First Affiliated Hospital of Chongqing Medical University, Chongqing, China; ^3^Department of the First Clinical Medicine, Chongqing Medical University, Chongqing, China; ^4^Department of Oncology, The First Affiliated Hospital of Chongqing Medical University, Chongqing, China

**Keywords:** psychiatry, attitude, career choice, medical students, psychiatry major

## Abstract

**Objectives:**

To understand the attitudes of medical students with a psychiatry major toward psychiatry at Chongqing medical university in China and to find out factors influencing students' career choice.

**Methods:**

The present study used an online web survey tool to assess the attitudes toward psychiatry amongst 422 students majoring in psychiatry at Chongqing medical university in China using sociodemographic and Attitudes Toward Psychiatry-30 items (ATP-30) scales. Descriptive statistics and logistic regression analysis were used to examine associated factors.

**Results:**

Three hundred and sixty-nine students (87%) answered the questionnaire. Nearly 54.5% of participants had overall positive attitudes to psychiatry and 80.8% thought psychiatrist could be a career choice. Of the students, 5.1% showed that they did not want to be a psychiatrist while the remaining 14.1% were undecided. The first and fifth year students showed less desire to be a psychiatrist (74.3 and 69.8%, respectively); the highest percentage recorded is of the third year (90.6%). Female participants, in contact with patients suffering from mental illness, were willing to study psychiatry as a master degree and see good prospects were positive factors in choosing psychiatry as a career.

**Conclusions:**

Students generally have good expectations toward psychiatry, but different opinions are also held and the field is still faced with various challenges in order to provide more psychiatrists.

## Introduction

Mental health is an essential component of health. Mental disorders can affect not only individual quality of life but also national productivity (1). Although the burden of mental illnesses has been increasing worldwide, such increases may be more dramatic in China (2), which can partly be understood as a consequence following rapid social changes in recent decades. Unfortunately, mental health services in China are insufficient to respond to the extensive mental disorders prevalent in the country. In 2004, the total number of licensed psychiatrists in China was 16,103 (1.24 psychiatrists per 100,000 people), with 557 psychiatric hospitals with 129,314 psychiatric beds (9.95 per 100,000) nationwide (3). In 2015, the number increased to 27,733 (2.01 psychiatrists per 100,000 people), and 2,936 mental health services with approximately 433,000 psychiatric beds (31.5 per 100,000) (4), but based on the World Health Organization (WHO)'s Mental Health Atlas (5), the proportion of psychiatrists in 2014 was about 20.1 per 100,000 in Japan.

There is a serious shortage of psychiatrists in China, therefore, the training of psychiatrists is of great importance, but many previous studies have consistently shown that psychiatry was not the top specialty choice for vocation among medical students in different countries (6–10). Although many factors, such as gender (11) and grades (12), will influence a medical student's career choice for psychiatry, attitudes about psychiatry play a key role (9, 10, 13). In previous studies, positive views on psychiatry, such as it being an intellectual challenge, and efficacy of psychiatry (14, 15) were seen, but there are some negative opinions associated with it, for instance, the lack of scientific foundation (16), low social status, relatively low income (14, 15), and poor personal image of psychiatrists in society (17). Generally speaking, whether or not to work as a psychiatrist is determined by complex factors and still worthy to study.

The Chongqing Medical University in Chongqing, China, was established in 1956, and provides psychiatry courses ranging from bachelor's to Master's Degree/Ph.D. In order to train more psychiatrists, the school established clinical medicine (mental hygiene specialty) in 2006. Ai investigated the employment situations of 390 students of clinical medicine (mental hygiene specialty) from grade 2006 to 2011 and found only 16.36% of them selected to be a psychiatrist (18). In 2016, the school introduced psychiatry as a major, which trained approximately 80 medical students majoring in psychiatry every year. Compared with undergraduate psychiatry education in the standard Chinese clinical medicine curriculum (CMC), medical students with a psychiatric major will take a psychiatry major curriculum (PMC), which provides more comprehensive exposure to psychiatry than CMC, with more preclinical experiences and psychiatry clerkship course hours. Nonetheless this does not mean all the students voluntarily select the PMC; there were 422 students majoring in psychiatry since 2016, but about 57.7% of them were placed into it from other majors upon admission to the university.

Our study explored the attitudes of psychiatry-majoring students in Chongqing medical school toward psychiatry. We conducted this research because: (1) Most studies are aimed at students of all majors. There are few studies on the attitudes of medical students with a psychiatry major, so it would be worthy to explore whether they would be more willing to be a psychiatrist; (2) we wished to explore what affects the choice of students when they face their career choice; and (3) Most of the studies are concentrated in the United States, the United Kingdom, Israel, and other countries. In western China, this type of study has not yet been found, so we hope to understand the attitude of students here.

## Methods

### Sample

An online sociodemographic questionnaire and Attitudes Toward Psychiatry-30 items (ATP-30) were sent to all students majoring in psychiatry (*n* = 422) at Chongqing Medical University, China. 369 (87%) students submitted the online questionnaire and gave full information.

### Design

The study consisted of a two-part questionnaire consisting of a self- administered scale and ATP-30, Chinese version. The ATP-30 was designed by Burra et al. (19). It was mainly used to measure the attitudes of medical students toward psychiatry, although now the scale has been widely used (20–22). ATP-30 focuses on attitudes and views of psychiatry with 30 questions. There are 5 choices for each question: strongly agree, agree, neutral, disagree, and strongly disagree. It contains 30 Likert-scale items, 5 means strongly agree and 1 means strongly disagree. These questions focus on many aspects of psychiatry, for example, the views on mental illness, psychiatrists, treatment, and on that of choosing to become a psychiatrist in the future. The total score ranged between 30 and 150 points. A score of 90 means “the logical neutral point of the scale,” scores more than 90 mean a more positive attitude (20). Students with a psychiatry major were invited to participate in the study from March 2021 to May 2021; there is no identity disclosure or financial compensation. Ethical approval was granted by the Chongqing Medical University, and informed consent was obtained from all participants.

### Statistical Analyses

We used “Tencent questionnaire” (an online web survey tool) to collect the data, and the statistical analysis was performed by SPSS version 25.0. We used descriptive statistics for counts (*n*), proportions (%), or means with standard deviations (SD). Multiple logistic regression analysis was used to find significant factors with the ATP-30 item 4 (I would like to be a psychiatrist), these factors include gender, income, and contact with psychiatric patients. Answers of item 4 were divided into 3 categories: “strongly agree,” “agree” were divided into the “agree” category, while “strongly disagree,” and “disagree” were divided into the “disagree” category, “neutral” remained as a category on its own. *P* < 0.05 was considered significant in this study.

## Results

Three hundred and sixty-nine students (87.4%, 369/422) finished both questionnaires, of which 106 were males (28.7%). Table 1 summarizes the demographic characteristics of the participants.

**Table 1:** Demographic Characteristics of participants (*n* = 369).

**Characteristic**	***N***	**%**
**Gender**		
Male	106	28.7
Female	263	71.3
**Have siblings**		
Yes	177	48
No	192	52
**Monthly total income of all the family members (RMB)**		
<3,000	84	22.8
3,000–8,000	142	38.5
>8,000	143	38.7
**Contact with psychiatric patients**		
Yes	184	49.9
No	185	50.1
**Willing to become a psychiatrist in the future**		
Yes	298	80.8
Neutral	52	14.1
No	19	5.1
**Willing to study for psychiatric master degree**		
Yes	298	80.8
Neutral	50	13.6
No	21	5.6
**Psychiatry as the first chosen major**		
Yes	156	42.3
No	213	57.7
**The prospects of the psychiatry major**		
Good	258	69.9
Neutral	92	24.9
Bad	19	5.2

Out of the 369 students who answered all the ATP-30 items, about 54.5% responded for positive attitudes to psychiatry, 39% of them showed generally negative attitudes, and 6.5% were neutral.

More females reported favorable attitudes to psychiatry compared to males (56.7% to 49%). More than 40% (48.7 to 43 to 41.3%) of students in the 1st, 2nd, and 5th year, respectively, reported negative attitudes. In contrast, to other grades, a higher percentage (more than 55%) of participants in their last 3 years of study showed positive attitudes to psychiatry (Table 2).

**Table 2:** Global ATP-30 scores by gender and grade (*n* = 369).

**Variable**	***n***	**Unfavorable**	**Neutral**	**Favorable**
		**ATP-30 <90 (%)**	**ATP-30 = 90 (%)**	**ATP-30 > 90 (%)**
**Gender**				
Male	106	45.3	5.7	49
Female	263	36.5	6.8	56.7
Total	369	39	6.5	54.5
**Grade**				
5th	63	41.3	3.2	55.5
4th	78	30.7	10.3	59
3rd	64	29.7	7.8	62.5
2nd	86	43	4.7	52.3
1st	78	48.7	6.4	44.9

The answers to item 4 of the ATP-30 (“I would like to be a psychiatrist”) were considered particularly significant because they provided the attitude of career choice in the future. The details of this item are presented in Table 3. In total, about 80.8% of the students expressed that they would choose to be a psychiatrist, but for the first year and the fifth year, the rate is relatively lower (74.3% and 69.8%); the highest one is the 3rd year (90.6%) (Table 3).

**Table 3:** “I would like to be a psychiatrist” in different grade (item 4 on ATP-30).

	**Rating** ***n*** **(%)**			
**Years of**	**Strongly**	**Agree**	**Neutral**	**Disagree**	**Strongly**
**study**	**agree**				**agree**
1	26 (33.3)	32 (41)	17 (21.8)	2 (2.6)	1 (1.3)
2	23 (26.7)	53 (61.6)	8 (9.3)	1 (1.2)	1 (1.2)
3	16 (25)	42 (65.6)	4 (6.3)	0	2 (3.1)
4	24 (30.8)	38 (48.7)	11 (14.1)	4 (5.1)	1 (1.3)
5	12 (19)	32 (50.8)	12 (19)	6 (9.5)	1 (1.6)

Regression analysis showed that gender (*p* = 0.041), contact with psychiatric patients (*p* = 0.003), those willing to study for a Master's Degree of psychiatry (*P* < 0.001), and good prospects in psychiatry (*P* < 0.001) were significantly associated with the wish to be a psychiatrist (Table 4).

**Table 4:** Multiple logistic regression model for “ATP item 4.”

**Variables**		**OR**	**95% CI**		***P***
**“I am willing to be a psychiatrist”**	Disagree	0.143	−.398	−0.498	0.008^*^
	Neutral	0.826	−1.598	1.215	0.79
	Agree	Ref			Ref
**Gender**	Male	0.502	−1.352	−0.028	0.041^*^
	Female	Ref			Ref
**Have siblings**	No	1.298	−0.417	0.938	0.451
	Yes	Ref			Ref
**Monthly total income of all the family members (RMB)**	<3,000	0.997	−0.833	0.828	0.995
	3,000–8,000	1.057	−0.674	0.784	0.883
	>8,000	Ref			Ref
**Contact with psychiatric patients**	No	0.978	−0.637	0.594	0.003^*^
	Yes	Ref			Ref
**Willing to study for master degree of psychiatry**	No	0.169	−2.867	−0.689	0.001^*^
	Neutral	1.42	−0.492	1.194	0.414
	Yes	Ref			Ref
**Psychiatry as the first chosen major**	No	1.195	−0.503	0.86	0.608
	Yes	Ref			Ref
**The prospects of the psychiatric major**	Good	10.095	1.093	3.532	0.001^*^
	Neutral	0.773	−1.32	0.804	0.634
	Bad	Ref			Ref

*Ref, reference group.*

* *P < 0.05*.

**Figure 1:**
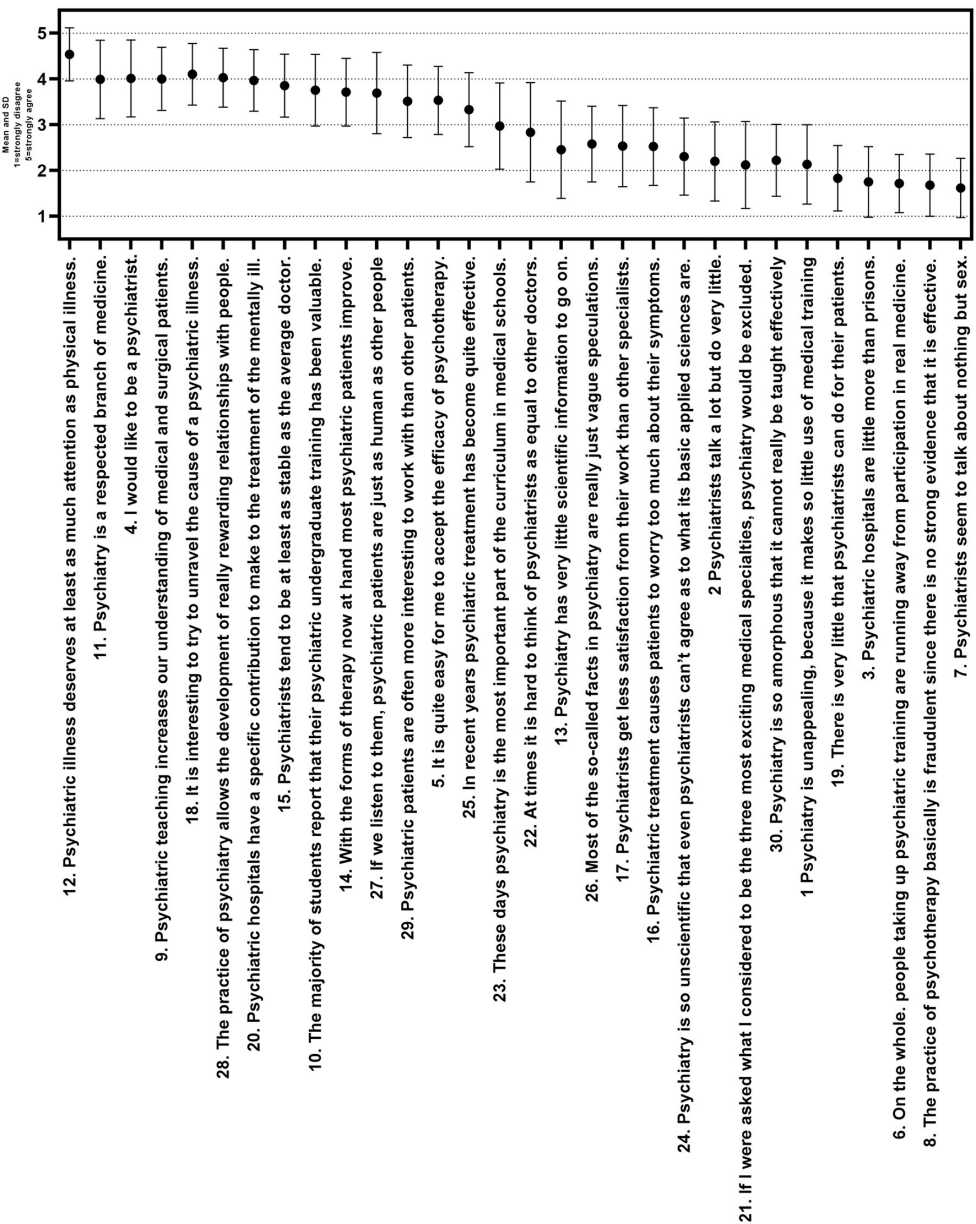
ATP-30 items according to the ATP-30.

From the results of ATP-30, in general, the students believe that psychiatry should be paid enough attention and respect. Psychiatry is interesting and psychiatric treatment is effective (item: 11, 12, 18). Most students disagree that there is a lack of science in psychiatry, or psychiatric treatment is cruel and ineffective, or that psychiatric treatment is useless (item: 2, 7, 19) [Figure 1: Mean and SD of the Attitudes Toward Psychiatry (ATP-30) items].

## Discussion

### Total Opinions From Students

Our results showed that 54.5% of students had positive attitudes to psychiatry and 80.8% expressed they would choose to be a psychiatrist. One study from 20 countries reported about 23.5% of students thought being a psychiatrist could be a “definitely or quite likely” career choice (24). Another study showed a dissonance between positive attitude (74.9%) and career choice (14.3%) to psychiatry (12). In our study, the proportion of wish to be a psychiatrist is rather high; this may be attributed to the following reasons: (1) the study focuses on the students of psychiatry major, who have a higher likelihood to have voluntarily selected to study psychiatry; (2) the 2020 Science and Technology Evaluation Metrics (STEM) of China's hospitals, which were based on the integration of production, teaching, and research, revealed the development of science and technology in medical colleges and universities in a comprehensive approach. It showed psychiatry in the first affiliated hospital of Chongqing Medical University ranked 6th among all the hospitals (25), furthermore, it is the only university providing psychiatric degree programs in Chongqing. The students may have more confidence in the major and have more optimistic expectations about being a psychiatrist in the future; and (3) we made a 5-year full tutorial system for students when they entered the program. The tutors would lead students to participate in clinical and scientific research work, and established a relatively strong interest in the early stage.

We noticed that the attitudes toward psychiatry in different grades are different; for the first-year group, students showed higher unfavorable attitudes, in fact, previous studies (16, 17) showed that the lower grades had a more positive attitude toward psychiatry than the higher grades, which was different from our study. Possible reasons may be the freshmen do not know much about psychiatry, and they may believe stereotypes regarding mental illness and the patients. Another possible reason may also be most of them do not voluntarily select the psychiatry major but are placed into psychiatry upon admission to the university.

A similar phenomenon was also found in the fifth-year group. This may be due to the fact that more factors will affect students' career choice as they approach graduation, such as low social status (14), relatively low income of psychiatry (15), the poor personal image of psychiatrists in society (17), and no opportunity to perform clinical skills (16). Similar results have been reported in Israel (23) and Pakistan (26).

Students showed the highest favorability to psychiatry during 3rd and 4th year; this may be because they begin their psychiatry exposure in these 2 years. The psychiatry major students in the Chongqing Medical University were introduced to clinical exposure to psychiatry (such as child and adolescent psychiatry, geriatric psychiatry, etc.) during the 3rd and 4th years. Some previous studies showed more positive attitudes after exposure to psychiatry (11, 27–29), while others showed opposite results (30, 31) or no effect (32). The difference may indicate that the effect of clinical exposure is influenced by complex factors.

### The Factors Impact the Career Choice

In our study, we found females had more favorable attitudes toward psychiatry and more willingness to be a psychiatrist. This result was similar to most previous studies (33, 34) but not to all (35, 36). The findings also showed more females would like to be a psychiatrist (30); the reason might be the so-called psycho-social disciplines by female students and doctors, which have been demonstrated in previous studies (37, 38). Another finding in our study is contact with mental illness patients might be a positive contributing factor to being a psychiatrist. This result is similar to many previous studies (39, 40), but opposite results are also found in other studies (16). One study found (24) that, compared with inpatients, contact with outpatients makes students more willing to choose to become a psychiatrist in the future, and the study also showed that contact with patients with mental illness during the recovery period will make students more willing to choose to become psychiatrists than contact with patients with acute mental illness. In our study, most of the psychiatric patients contacted by students are relatives or friends who have more positive emotion and symptoms which are not so severe. This may be the reason for the differences in this study. Willingness to study for a master's degree in psychiatry and have good prospects of psychiatry are also positive factors to be a psychiatrist in the future. There are also related studies in the past (15), suggesting that better expectations for psychiatry indicate a greater chance of choosing to become a psychiatrist in the future.

### Individual Questions From the ATP-30

The results shown in Figure 1 provide some information for different factors that lead to both negative and positive attitudes. The attitudes of medical students are generally quite positive. Among notable positive attitudes, students mostly see psychiatry as a valid branch of medicine, the treatment of psychiatry is effective, and patients really get cured by modern treatments. A majority of the students holding negative opinions are mainly due to the low professional achievement of psychiatrist, where they think psychiatric hospitals are like prisons, or psychiatrists always talk about sex, which is still a topic of taboo in China.

For instance, 19.8% of students agreed that psychiatry has very little scientific information to go on, pointing out a possible poor scientific view for psychiatry. This view has been shown many times in different studies (16, 23); this is also one of the most controversial questions. To the question, 16% strongly disagreed, 46.1% disagreed, 18.2% were neutral, 15.7% agreed, and 4.1% strongly agreed. One possible reason might be the psychiatric diagnosis was not based on physical findings or laboratory tests but mainly the symptoms.

Another controversial statement in the questionnaire was probably the one that “Psychiatrists get less satisfaction from their work than other specialists.” To the question, 10% strongly disagreed, 48.2% disagreed, 31.4% were neutral, 15.2% agreed, and 0.5% strongly agreed. This question may involve complex factors, such as views about low status, the patient's treatment effect, income, and others (14–16).

Questions like “Psychiatrists seem to talk about nothing but sex (item 7)” and “Psychiatric hospitals are little more than prisons (item 3),” shows significant stigmatization of patients, psychiatrists, and psychiatry. This stigmatization has also been found in many studies, and it may cause students to give up working in psychiatric-related fields after graduation (11, 12, 38). However, in our study, most students disagreed with these views. Possible reasons may be when students enter the school in their freshman year, the teachers will schedule hold lectures and comprehensively introduce all aspects of psychiatry, which may involve the development of psychiatric treatment, psychiatry used in daily life, and the benefit of psychiatry for individuals. This could cultivate students' enthusiasm for psychiatry and eliminate misunderstandings about psychiatry. At the same time, every semester, there will be 3–4 weeks for students to visit wards and outpatient clinics and the students will have preclinical experience (41), but even so, mental illness patients and psychiatric hospitals are misunderstood by the general public, so it still requires more correct guidance from society and the country.

This study also has some limitations: (1) it focuses on the attitudes of psychiatry major students, but not all medical students in Chongqing Medical University. In the following study, we will include more participants from different specialties; and (2) this study contains limited factors, for example, research on teacher-student relationship, teaching methods, and other related factors that can be included in the following study.

## Conclusion

This study found that the majority of students endorsed favorable attitudes toward psychiatry, while gender and personal patient contact clearly influence students' attitudes. Willingness to take a master's degree study of psychiatry, as well as good prospects of psychiatry, appear to be positive factors influencing students' attitudes to be psychiatrists. In order to counteract the lack of medical students becoming psychiatrists, it is important to provide more preclinical chances for students, and the country should also provide more positive guidance on psychiatry.

## Data Availability Statement

The raw data supporting the conclusions of this article will be made available by the authors, without undue reservation.

## Ethics Statement

The studies involving human participants were reviewed and approved by the Ethics Committee of the Chongqing Medical University. The patients/participants provided their written informed consent to participate in this study.

## Author Contributions

XL conceived the structure of the manuscript and wrote the manuscript. XC collected the data. YG, JH, and YT analyzed the data. JZ, HY, MA, and LK critically reviewed the manuscript. All authors have read and approved the final manuscript.

## Funding

This research was supported by the National Natural Science Foundation of China (No: 81971286), the Chongqing Higher Education Teaching Reform Research Project (Grant No.: 171012), Education and Teaching Research Project of Chongqing Medical University (Grant No.: JY170101), and the Chongqing Health Commission (Grant No.: 2020FYYX159; Grant No.: 2022MSXM058). The funding body had no involvement in study design, in the collection, analysis, and interpretation of data, in the writing of the report, and in the decision to submit the article for publication.

## Conflict of Interest

The authors declare that the research was conducted in the absence of any commercial or financial relationships that could be construed as a potential conflict of interest.

## Publisher's Note

All claims expressed in this article are solely those of the authors and do not necessarily represent those of their affiliated organizations, or those of the publisher, the editors and the reviewers. Any product that may be evaluated in this article, or claim that may be made by its manufacturer, is not guaranteed or endorsed by the publisher.
